# Fasting plasma chenodeoxycholic acid and cholic acid concentrations are inversely correlated with insulin sensitivity in adults

**DOI:** 10.1186/1743-7075-8-48

**Published:** 2011-07-07

**Authors:** Bertrand Cariou, Maud Chetiveaux, Yassine Zaïr, Etienne Pouteau, Emmanuel Disse, Béatrice Guyomarc'h-Delasalle, Martine Laville, Michel Krempf

**Affiliations:** 1 INSERM, UMR915; Université de Nantes; CHU Nantes, Clinique d'Endocrinologie, Maladies Métaboliques et Nutrition, l'Institut du Thorax, Nantes, CRNH Nantes, F-44000 France; 2 Nestlé Research Center, R&D Santiago, 9260075 Maipú Santiago, Chile; 3 CRNH Rhone-Alpes; INSERM Unit -1060, CarMeN Laboratory and CENS, Lyon1 University; Hospices Civils de Lyon, France Centre Hospitalier Lyon-Sud, F-69310 Pierre Bénite, France

**Keywords:** bile acids, insulin resistance, type 2 diabetes, FXR, TGR5, energy expenditure, hyperinsulinemic-euglycemic clamp

## Abstract

**Background:**

Accumulating data suggest a novel role for bile acids (BAs) in modulating metabolic homeostasis. BA treatment has been shown to improve glucose tolerance and to increase energy expenditure in mice. Here, we investigated the relationship between fasting plasma BAs concentrations and metabolic parameters in humans.

**Findings:**

Fasting plasma glucose, insulin and lipid profile were measured in 14 healthy volunteers, 20 patients with type 2 diabetes (T2D), and 22 non-diabetic abdominally obese subjects. Insulin sensitivity was also assessed by the determination of the glucose infusion rate (GIR) during a hyperinsulinemic-euglycemic clamp in a subgroup of patients (9 healthy and 16 T2D subjects). Energy expenditure was measured by indirect calorimetry. Plasma cholic acid (CA), chenodeoxycholic acid (CDCA) and deoxycholic acid (DCA) concentrations were analyzed by gas chromatograph-mass spectrometry. In univariable analysis, a positive association was found between HOMA-IR and plasma CDCA (β = 0.09, p = 0.001), CA (β = 0.03, p = 0.09) and DCA concentrations (β = 0.07, p < 0.0001). Spearman analysis retrieved an inverse relationship between plasma CDCA (r = -0.44, p = 0.03), CA (r = -0.65, p = 0.001) and the GIR. HOMA-IR remained positively associated with CDCA (β = 0.11, p = 0.01), CA (β = 0.04, p = 0.01) and DCA (β = 0.06, p = 0.007) in multivariable analysis, after adjustment for age, gender, BMI, HbA1C and plasma lipid parameters. In contrast, HbA1c, energy expenditure and plasma lipid concentrations were not correlated with plasma BAs levels in multivariable analysis.

**Conclusions:**

Both plasma CDCA, CA and DCA concentrations were negatively associated with insulin sensitivity in a wide range of subjects.

## Background

Bile acids (BAs) and BA receptors emerge as new modulators of glucose homeostasis [1]. FXR (farnesoid × receptor) is a nuclear receptor that controls BA and lipid metabolism [2,3]. Several studies have highlighted an unexpected role of FXR in metabolic homeostasis [4,5]. While FXR-deficient mice exhibit insulin resistance [6,7], treatment of *ob/ob *and *db/db *diabetic mice with a synthetic FXR agonist improves blood glucose levels as well as insulin sensitivity [6,8]. However, the picture is more complex since FXR deficiency improves glucose homeostasis and attenuates body weight gain in an obese genetic background [9].

The addition of cholic acid (CA) to the diet increases energy expenditure and prevents the development of high fat-induced obesity and insulin resistance in mice [10]. This metabolic effect of CA is mediated by the binding to the G-protein-coupled receptor TGR5 [11,12], leading to the induction of the cAMP-dependent thyroid hormone activation enzyme DIO2 (type 2 iodothyronine deiodinase) in brown adipose tissue [10]. Human TGR5 is activated by several BAs, with lithocholic acid (LCA) and deoxycholic acid (DCA) being the most potent natural agonists [12]. In addition, the activation of TGR5 by a semi-synthetic specific agonist induces GLP-1 secretion and improves glucose tolerance in mice [13].

The aim of the present study was to ascertain whether plasma BA concentrations are linked to insulin sensitivity, assessed both by the homeostasis model assessment (HOMA-IR) and the gold-standard hyperinsulinemic-euglycemic clamp method, in a variety of subjects including healthy volunteers, obese and type 2 diabetic (T2D) patients.

## Methods

### Subjects

The baseline characteristics of the 56 participants involved in the study are presented in Table 1. The study group consisted of 14 healthy lean volunteers, 22 obese subjects and 20 patients with T2D. All the participants had no acute pathology at the time of the clamp. Healthy volunteers and obese subjects did not take any medication. T2D patients were under diet only or had discontinued their anti-diabetic medications, but not hypolipidemic drugs, at least 4 weeks before the clamp. They were asked to consume a standardized isocaloric diet and to maintain a normal physical activity during the 3 days before the study. The patients were recruited in two centers: Human Nutrition Research Center of Nantes (*Center 1*) and Rhône-Alpes (*Center 2*). All participants gave their written consent after being informed of the nature, purpose, and possible risks of the study. The experimental protocols were conducted before July 1, 2008, and were approved by the ethical committees of the CHU de Nantes and Hospices Civils de Lyon and performed according to French legislation.

**Table 1:** Anthropometric, biochemical, and metabolic parameters of the studied groups

	Control	Obese	T2D
n	14	22	20
Age (yr)	45 [41;59]	49 [20;60]	55 [40;63]^#,$^
Gender (males/females)	5/9	20/2	12/8
BMI (kg/m2)	23.85 [21.20;32.40]	33.60 [27.90;38.50] *	31.85 [22.80;39.10]^#^
Fasting glucose (mg/dl)	5.03 [4.50;5.85]	5.50 [4.30;6.80] *	7.37 [5.11;12.50]^#,$^
HbA1c (%)	5.40 [5.00;5.90]	5.45 [4.80;6.50]	7.40 [5.80;11.70]^#,$^
Fasting insulin (μUI/ml)	5.90 [2.90;13.90]	11.55 [3.90;35.70] *	11.65 [2.10;38.40]^#^
HOMA	1.33 [0.60;3.03]	3.15 [0.85;10.79]	3.85 [0.66;16.38]^#^
BMR (kcal/24 h)	1368 [1066;1920]	1895 [1590;2350] *	1701 [1264;2062]^#,$^
TC (mg/dl)	209 [170;292]	201[138;250]	184 [104;219]^#^
TG (mg/dl)	90 [51;187]	140 [62;531] *	130 [44;335]
LDL-C (mg/dl)	122 [86;178]	131 [92;165]	112 [35;139]^#,$^
HDL-C (mg/dl)	66 [40;103]	40 [26;54] *	46 [37;74]^#,$^
Total BA (μmol/l)	1.06 [0.46;1.90]	1.49 [0.64;4.06]*	1.34 [0.53;5.54]
DCA (μmol/l)	0.38 [0.14;1.02]	0.44 [0.03;1.07]	0.62 [0.11;1.66]^#^
CDCA (μmol/l)	0.36 [0.09;1.25]	0.60 [0.31;2.38]	0.43 [0.09;2.78]
CA (μmol/l)	0.22 [0.08;0.50]	0.35 [0.09;1.20] *	0.26 [0.1;1.10]

Data represent median [min;max]. Total BA represent the sum of DCA + CDCA + CA.

Tukey test was used for comparison of clinical parameters between the groups. Statistically significant differences (p < 0.05) are shown between controls and obeses (*), control and type 2 diabetics (^#^), and obeses and type 2 diabetics (^$^).

HOMA: homeostasis model assessment (= glucose × insulin/22.5), BMR: basal metabolic rate, TC: total cholesterol, TG: triglycerides, DCA: deoxycholic acid, CDCA: chenodeoxycholic acid, CA: cholic acid.

### Study design

The study was conducted after a 12-h overnight fast. Fasting plasma glucose and insulin were determined by the glucose oxidase method (Glucose HK, Roche Diagnostics, Meylan, France) and radioimmunoassay (bi-insulin IRMA, Cisbio international, Gif-sur-Yvette, France). HbA1c was measured by HPLC (TOSOH, A1C2.2 - G5). Plasma CA, CDCA and DCA concentrations were analyzed by gas chromatograph-mass spectrometry according to the method of Stellaard F. *et al*. [14]. Insulin sensitivity was also measured with the hyperinsulinemic-euglycemic clamp technique according to DeFronzo *et al *[15]. Insulin infusion rate was 40 mU/m^2^. min in the *Center 1 *and 75 mU/m^2^. min in the *Center 2*. Energy expenditure was continuously measured during 30 min throughout the test by open circuit indirect calorimetry (Deltatrac II, Datex Instrument, Helsinki, Finland) under basal supine condition, before performing the clamp. The average gas exchange recorded over the 30-min period was used to calculate the basal metabolic rate.

### Statistical analysis

Continuous variables are expressed as means and standard deviation and categorical variables as percent and count. Analysis of variance (ANOVA) or Kruskal-Wallis test was used for comparison of the clinical parameters among 3 groups. If ANOVA showed a significant difference between the groups, Tukey test was used for comparison of clinical parameters between two groups. Categorical variables were compared by chi-square test. Linear simple and multiple regression analyses were performed for estimation of association between plasma bile acids concentrations and metabolic parameters. All factors were included in linear multiple regression analyses. Spearman coefficients were used for correlation between plasma BA levels and GIR. A *p *value ≤ 0.05 was considered statistically significant. Statistical analysis was performed with SAS for Windows version 9.1 software (SAS Institute Inc, Cary, NC, USA).

## Results

### Circulating bile acids and glucose homeostasis

Total plasma BAs concentrations were higher in both obese and T2D in comparison with the healthy controls, but the difference reached statistical significance only for the obese subjects compared to controls (Table 1). Plasma DCA was significantly higher in T2D compared to control group, while plasma CA was significantly increased in obese patients compared to controls.

**Figure 1:**
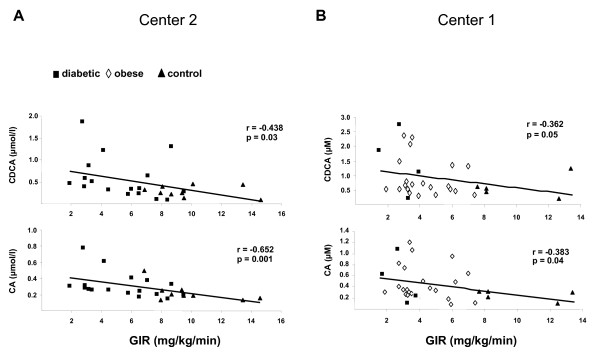
**Spearman correlation of plasma bile acids concentrations and insulin sensitivity in healthy controls (n = 14) (black triangle), type 2 diabetic (black square) (n = 20) and obese (white diamond) (n = 22) subjects**. Plasma chenodeoxycholic acid (CDCA) and cholic acid (CA) concentrations were measured by GC-MS analysis. Insulin sensitivity was assessed by the rate of infused glucose necessary to maintain euglycaemia during infusion of insulin (GIR: glucose infusion rate). In the center 2 (**A**), the insulin infusion rate was 75 mU/m^2^, while it was 40 mU/m^2 ^in the center 1 (**B**).

Linear simple regression analyses (Table 2) indicated that plasma BAs concentrations negatively correlated with insulin sensitivity. Indeed, DCA, CDCA and CA plasma concentrations were positively associated with fasting plasma insulin levels and HOMA-IR. To further assess insulin sensitivity with the gold standard method, we measured the GIR during a hyperinsulinemic-euglycemic clamp. Importantly, the GIR was inversely associated with plasma CDCA (r = -0.36, p = 0.05 for *Center 1*; r = -0.44, p = 0.03 for *Center 2*) and CA (r = -0.38, p = 0.04 for *Center 1*; r = -0.65, p = 0.001 for *Center 2*) levels; while the negative correlation with the DCA did not reach statistical significance (r = -0.30, p = 0.10 for *Center 1*; r = -0.35, p = 0.08 for *Center 2*) (Figure 1A-B). Except for a positive correlation between DCA and fasting blood glucose levels, no association was detected between BAs and the glycaemic status (Table 2).

**Table 2:** Linear simple regression analysis on plasma bile acid concentrations and variables

	DCA			CDCA			CA		
	
	*β*	95% confidence interval	p-value	*β*	95% confidence interval	p-value	*β*	95% confidence interval	p-value
**Age**	0.01	[0.002;0.02]	0.02	0.001	[-0.02;0.02]	NS	0.004	[-0.003;0.01]	NS
**Men**	0.002	[-0.19;0.19]	NS	0.39	[0.04;0.74]	0.03	0.17	[0.03;0.31]	0.02
**BMI**	0.02	[0.0006;0.04]	0.04	0.04	[0.004;0.07]	0.03	0.01	[-0.003;0.03]	NS
**Fasting glucose**	0.8	[0.03;0.12]	0.001	0.05	[-0,04;0.14]	NS	0.03	[-0.03;0.04]	NS
**Fasting insulin**	0.02	[0.01;0.03]	< 10^-4^	0.04	[0.02;0.06]	0.001	0.02	[0.006;0.02]	0.001
**HbA1C**	0.04	[-0.03;0.12]	NS	-0.05	[-0.18;0.08]	NS	-0.04	[-0.09;0.02]	NS
**HOMA-R**	0.07	[0.04;0.19]	< 10^-4^	0.09	[0.04;0.14]	0.001	0.03	[0.007;0.05]	0.09
**BMR**	0.0001	[-0.0002;0.0004]	NS	0.0008	[0.0002;0.001]	0.01	0.0002	[2.4*10^-7 ^;0.0005]	0.05

Linear simple regression analyses were performed for estimation of association between plasma BAs concentrations and metabolic parameters. A *p *value ≤ 0.05 was considered statistically significant.

To further assess the strength of the correlation between circulating BAs levels and insulin sensitivity, a multiple regression analysis was performed (Table 3). The positive correlation of plasma BAs concentrations with HOMA-IR remained significant after adjustment for each categorical variable: CDCA (β = 0.116, p = 0.01), CA (β = 0.045, p = 0.01) DCA (β = 0.062, p = 0.007). In contrast, plasma BAs levels were neither associated with fasting plasma glucose nor the HbA1c.

**Table 3:** Linear multiple regression analysis on plasma bile acids concentrations and variables

	CDCA		
	
	*β*	95% confidence interval	p-value
**Center**			NS
**- Center 2**	1		
**- Center 1**	0.466	[-0.037;0.969]	
**Group**			NS
**-Controls**	1		
**-Obeses**	0.144	[-0.614;0.902]	
**-Diabetics**	0.259	[-0.534;1.053]	
**Age**	-0.001	[-0.019;0.018]	NS
**BMI**	-0.013	[-0.066;0.039]	NS
**HbA1C**	-0.044	[-0.242;0.155]	NS
**HOMA**	0.116	[0.035;0.196]	0.01
**BMR**	-0.00007	[-0.0009;0.0007]	NS
**TG**	0.129	[-0.129;0.387]	NS
**LDL-C**	-0.133	[-0.961;0.695]	NS

	**CA**		
	
	***β***	**95% confidence interval**	**p-value**

**Group**			NS
**-Controls**	1		
**-Obeses**	0.350	[0.013;0.687]	
**-Diabetics**	0.164	[-0.189;0.517]	
**Age**			0.04
**-Center 2**	-0.008		
**-Center 1**	0.009		
**BMI**	-0.02	[-0.038;0.005]	NS
**HbA1C**	-0.015	[-0.093;0.063]	
**HOMA**	0.045	[0.014;0.077]	0.01
**BMR**	-0.0003	[-0.0006;0.0001]	NS
**TG**	0.053	[-0.048;0.155]	NS
**LDL-C**	-0.162	[-0.487;0.164]	NS

	**DCA**		
	
	***β***	**95% confidence interval**	**p-value**

**Center**			NS
**- Center 2**	1		
**- Center 1**	0.176	[-0.102;0.454]	
**Group**			NS
**-Controls**	1		
**-Obeses**	-0.067	[-0.486;0.352]	
**-Diabetics**	0.229	[-0.204;0.662]	
**Age**	0.007	[-0.003.0.017]	NS
**BMI**	0.004	[-0.025.0.033]	NS
**HbA1C**	-0.064	[-0.169;0.041]	NS
**HOMA**	0.062	[0.018.0.105]	0.007
**BMR**	0.00001	[-0.0004;0.00048]	NS
**TG**	-0.004	[-0.146;0.137]	NS
**LDL-C**	0.007	[-0.411;0.425]	NS

Linear multiple regression analyses were performed for estimation of association between plasma bile acids concentrations and metabolic parameters.

All variables (center, group, gender, age, BMI, HbA1c, HOMA, GIR, BMR, TG, LDL-C) were included in linear multiple regression analyses.

A *p *value ≤ 0.05 was considered statistically significant.

### Circulating bile acids and energy expenditure

A slight positive correlation was found between plasma CDCA and CA concentrations and the basal metabolic rate (BMR) in simple regression analysis (Table 2). However, plasma BAs concentrations were not found to be independently associated with BMR in multiple regression analysis (Table 3). Although a significant positive relationship was identified between plasma DCA and CDCA levels and BMI in simple regression, no further significant association between the BMI and plasma BAs levels was found in multiple regression analysis.

## Discussion

Besides their role as detergent molecules, BAs appear to modulate a broad spectrum of metabolic pathways including lipid and glucose homeostasis [1]. These findings are mainly based on animal studies and the clinical impact of BAs on metabolic homeostasis remains to be determined in human. Here, we show that plasma CDCA, CA and DCA concentrations were negatively associated with insulin sensitivity in a variety of subjects, including healthy volunteers, obese and T2D patients. In contrast, plasma levels of these BAs were not correlated with glycaemic status. Importantly, HOMA-IR remained positively related to those plasma BAs levels in multivariable analysis. When assessing insulin sensitivity by the clamp procedure, we further observed an inverse relationship between plasma CDCA and CA concentrations and insulin sensitivity (*i.e*. GIR).

Our data sustain the hypothesis for a role of plasma BAs and FXR in insulin resistance. FXR-deficient mice, which exhibit high plasma BA concentrations [2,3], display impaired peripheral insulin sensitivity [6,7]. Conversely, treatment with synthetic specific FXR agonists improved insulin sensitivity in rodents [6,8], and in patients with non-alcoholic fatty liver disease [16]. Previous studies have suggested a link between BA pool size and glucose metabolism in humans. In a study performed in 6 Pima Indians with T2D, the BA pool was found to be increased during untreated hyperglycaemia and conversely decreased upon insulin therapy [17]. In contrast, the BA pool size remained unaltered upon insulin treatment in another study involving 14 patients with T2D [18]. Very recently, a well-designed study has retrieved an increase in the DCA absolute pool size, without change in total pool size in patients with T2D [19]. Accordingly, plasma DCA levels were significantly increased in T2D patients in comparison with controls in the present study. In lipid-lowering trials, BA sequestrants, which disrupt the enterohepatic circulation of BAs, have been shown to lower plasma glucose and HbA1c [20]. However, the changes in BA metabolism following colesevelam treatment were not related to changes in glucose metabolism in patients with T2D [19]. In contrast, a metabolomic study in healthy and pre-diabetic individuals found a correlation between plasma BAs levels and insulin sensitivity [21]. Thus, additional studies using FXR or TGR5 agonists are needed to unravel the effect of BAs on insulin sensitivity in human.

In mice, plasma BAs increase energy expenditure *via *a TGR5-mediated signalling cascade in brown adipose tissue [10]. This pathway has been suggested to operate in human skeletal myocytes [10], but its clinical relevance has been challenged recently [22]. Here, we found a slight positive correlation between CDCA and CA plasma levels and energy expenditure. In contrast, plasma levels of DCA, one of the best ligand of TGR5 [12], did not associate with BMR. Moreover, circulating BAs failed to correlate with energy expenditure in multivariable regression analysis. One limitation of our study is that all the components of the plasma BA profile were not analyzed. Thus, we can not exclude that some low abundant plasma BA species (for instance, LCA) might affect insulin sensitivity or energy expenditure.

In summary, we found that plasma CDCA and CA, and in a lesser extent DCA, were related to insulin resistance in a wide range of subjects. These data sustain the hypothesis for a role of BAs in glucose homeostasis.

## List of abbreviations

BA: bile acid; BMI: body mass index; BMR: basal metabolic rate; CA: cholic acid; CDCA: chenodeoxycholic acid; DCA: deoxycholic acid; DIO-2: type 2 iodothyronine deiodinase; FXR: farnesoid × receptor; GIR: glucose infusion rate; GLP-1: glucagon-like peptide 1; HOMA: homeostasis model assessment; LCA: lithocholic acid; T2D: type 2 diabetes.

## Competing interests

The authors declare that they have no competing interests.

## Authors' contributions

BC conceived of the study, participated in its design and coordination and drafted the manuscript. MC performed the dosage of BAs. YZ, ED performed the clamps. BGD performed the statistical analysis. EP, ML and MK contributed to the analysis of the data and critical revision of the manuscript. All authors read and approved the final manuscript.
